# Status and Prospects of Robotic Gastrectomy for Gastric Cancer: Our Experience and a Review of the Literature

**DOI:** 10.1155/2017/7197652

**Published:** 2017-05-23

**Authors:** Sejin Lee, Taeil Son, Hyoung-Il Kim, Woo Jin Hyung

**Affiliations:** ^1^Department of Surgery, Graduate School, Yonsei University College of Medicine, Seoul, Republic of Korea; ^2^Department of Surgery, Yonsei University College of Medicine, Seoul, Republic of Korea; ^3^Gastric Cancer Center, Yonsei Cancer Center, Yonsei University Health System, Seoul, Republic of Korea; ^4^Robot and MIS Center, Severance Hospital, Yonsei University Health System, Seoul, Republic of Korea

## Abstract

Since the first report of robotic gastrectomy, experienced laparoscopic surgeons have used surgical robots to treat gastric cancer and resolve problems associated with laparoscopic gastrectomy. However, compared with laparoscopic gastrectomy, the superiority of robotic procedures has not been clearly proven. There are several advantages to using robotic surgery for gastric cancer, such as reduced estimated blood loss during the operation, a shorter learning curve, and a larger number of examined lymph nodes than conventional laparoscopic gastrectomy. The increased operation time observed with a robotic system is decreasing because surgeons have accumulated experience using this procedure. While there is limited evidence, long-term oncologic outcomes appear to be similar between robotic and laparoscopic gastrectomy. Robotic procedures have a significantly greater financial cost than laparoscopic gastrectomy, which is a major drawback. Recent clinical studies tried to demonstrate that the benefits of robotic surgery outweighed the cost, but the overall results were disappointing. Ongoing studies are investigating the benefits of robotic gastrectomy in more complicated and challenging cases. Well-designed randomized control trials with large sample sizes are needed to investigate the benefits of robotic gastrectomy compared with laparoscopic surgery.

## 1. Introduction

Minimal invasive surgery is considered to be the alternative standard for treating early gastric cancer. Previous studies have demonstrated benefits of minimal invasive surgery compared with open surgery with respect to postoperative pain, hospital stay, gastrointestinal function recovery, and return to normal activity [1–5]. In addition to postoperative outcomes, recent multicenter prospective randomized clinical trials showed that treating early gastric cancer with laparoscopic gastrectomy was sufficiently safe in terms of oncological aspects and could be established as the standard procedure in clinical practice [6]. However, some problems have not been solved, such as D2 lymph node dissection, anastomosis technique, and oncological safety in advanced gastric cancer. To overcome these challenges, surgeons investigated new techniques and instruments for the treatment of gastric cancer.

The da Vinci® Surgical System (Intuitive Surgical Inc., Sunnyvale, CA, USA) was developed in 1988. This robotic system was designed to address problems related to laparoscopic surgery. Since the robotic gastrectomy procedure was introduced [7], experienced laparoscopic surgeons have used this surgical system to treat gastric cancer. Recent reports demonstrated that robotic gastrectomy was a safe and feasible alternative treatment for gastric cancer compared with conventional laparoscopic gastrectomy [8–10]. However, to date, there are no well-designed prospective randomized trials investigating robotic gastric cancer surgery. In this review, we describe the current state of the field and its prospects for the treatment of gastric cancer.

## 2. Overview and History

By 2015, 3597 da Vinci Surgical Systems had been installed worldwide and used in approximately 650,000 procedures, usually for urological, gynecological, and general surgical procedures. The first robotic gastrectomy was reported in 2003 [7], and surgeons in the field of minimally invasive surgery adopted this new surgical approach as an experimental procedure for the treatment of gastric cancer. Robotic surgical systems offer several advantages, including high-resolution 3-D images, an EndoWrist® with seven degrees of freedom, and tremor filtering. Thus, this technique was expected to increase the accuracy and thoroughness of minimally invasive gastrectomy [11, 12].

Our institution also hoped that the unique advantages of robotic surgery could help with overcoming the limitations of laparoscopic surgery in gastric cancer treatment. In 2005, we adopted the robotic surgical system for the treatment of gastric cancer and have performed the largest numbers thereof in this field. Initial applications were limited only to early gastric cancer; however, this has been expanded alongside the expansion of indications for minimally invasive surgery. Regardless of the issues related to this new technology, including its tremendous financial cost, subjective perceptions of proficiency during the procedure among surgeons and some noted benefits in postoperative outcomes seem to have encouraged our surgeons to continue with performing robotic gastrectomy.

In the literature, several investigational case series (Table 1) and retrospective studies (Table 2) investigated robotic gastrectomy compared with conventional gastrectomy for the treatment of gastric cancer. Recently, Wang et al. reported a single-center randomized clinical trial for robotic gastrectomy compared with open gastrectomy [13]. This report showed that robotic gastrectomy group had less blood loss (94.2 ± 51.5 versus 152.8 ± 76.9 ml, *p* < .001), a shorter hospital stay (5.6 ± 1.9 versus 6.7 ± 1.9 days, *p* = .021), and earlier recovery of bowel function (2.6 ± 1.1 versus 3.1 ± 1.2 days, *p* = .028), but a longer operation time (242.7 ± 43.8 versus 192.4 ± 31.5 min, *p* = .002), than the open gastrectomy group. The complication rate and number of retrieved lymph nodes were not different between the two groups. Most comparative studies compared short-term surgical outcomes between robotic and laparoscopic gastrectomy. However, to our knowledge, there are no well-designed randomized trials investigating robotic gastrectomy compared with conventional laparoscopic gastrectomy. One large nonrandomized multicenter prospective study comparing robotic and laparoscopic gastrectomy in Korea was recently reported. The results showed that perioperative surgical outcomes of robotic gastrectomy were not superior to those of laparoscopic gastrectomy [14].

## 3. Issues regarding Robotic Gastrectomy

### 3.1. Operation Time

Previous comparative studies observed a longer operation time for robotic gastrectomy compared with that for conventional laparoscopic gastrectomy, although the statistical significance of the difference varied between studies. One of the major factors prolonging operation time in robotic surgery was docking and additional preoperation time [15, 16]. In addition, the initial learning curve of a procedure can also prolong operation time [17]. Eom et al. suggested that a robotic operating system, in which the surgeon alone performs the roles of operator, assistant, and camera operator, could contribute to the lengthy operation time for robotic gastrectomy [18]. However, a recent report on robotic gastrectomy showed that the operation time of the later 100 cases was significantly shorter than that of the earlier 100 cases [19]. Huang et al. also observed that operation time and docking time reduced significantly after completing a learning curve of 25 procedures [20]. A comparative study of robotic and laparoscopic gastrectomies performed by one experienced surgeon showed that the total operation time using robotic system was not statistically longer than that of laparoscopy [21]. Clearly, operation time decreases as the surgeon gains experience with the robotic system.

### 3.2. Blood Loss

Previous studies reported that robotic gastrectomy produced less estimated blood loss than conventional gastrectomy [10, 12, 22–26]. In addition, recent meta-analyses comparing robotic and laparoscopic gastrectomy showed that there was a trend toward reduced blood loss when a robotic system was used [27, 28]. Reduced blood loss can be attributed to the three-dimensional view and EndoWrist function found in robotic systems. Notably, these results were achieved early in the learning curve [29]. However, the level of evidence included in these systematic reviews was not high enough to draw strong conclusions due to a lack of randomized trials. For example, one study reported increased blood loss early in their experience with a robotic system [18]. A recent multicenter nonrandomized prospective comparative study of robotic versus laparoscopic gastrectomy for gastric cancer showed that estimated blood loss was similar between the two groups [14]. A subgroup analysis of that study showed that the robotic group had significantly lower estimated blood loss than the laparoscopic group during D2 lymph node dissection [30]. Lee et al. reported that in terms of blood loss, the benefits of a robotic approach were more apparent for high BMI patients when performing a distal gastrectomy with D2 lymph node dissection [25]. The reduction of blood loss probably has little impact on immediate patient outcomes. However, it may have oncological benefits because it could minimize the dissemination of free cancer cells during gastrectomy for cases of advanced gastric cancer, which is not a trivial concern [31–33].

### 3.3. Length of Hospital Stay

Compared with open gastrectomy, robotic gastrectomy had a shorter length of postoperative hospital stay [10, 13]. However, most studies showed no difference in length of hospital stay when comparing robotic and laparoscopic gastrectomies. This result could be due to the similar number of trocars used and slightly longer operation times required for robotic gastrectomy [11, 18, 22–26, 34–36]. Meanwhile, several reports showed shorter hospital stays for patients undergoing robotic gastrectomy compared with those undergoing laparoscopic surgery [6, 37–39]. Suda et al. reported that postoperative hospital stay was 14 days in the robotic gastrectomy group and 15 days in the laparoscopic gastrectomy group (*p* = .021) [40]. Similarly, Kim et al. reported shorter hospital stays for robotic surgery (6.7 ± 1.0 versus 7.4 ± 2.4 days in the laparoscopic surgery group, *p* < .001) [6]. In this report, the shorter hospital stay could be due to the relatively younger age of the robotic gastrectomy group (54.1 ± 12.0 versus 60.5 ± 11.0 years in the laparoscopic surgery group, *p* < .001). An age-controlled comparative study showed no statistical difference in hospital stay between patients of various ages undergoing robotic or laparoscopic gastrectomy (robotic gastrectomy in elderly patients; age 74.8 ± 4.8; hospital stay, 5 days versus robotic gastrectomy in young patients; age 51.1 ± 10.2; hospital stay, 5 days versus laparoscopic gastrectomy in elderly patients; age 73.1 ± 3.7; hospital stay, 6 days) [36]. Based on the postoperative stay results, it is unclear whether robotic gastrectomy has an advantage with respect to postoperative recovery.

### 3.4. Cost

The use of a robotic system to treat certain diseases remains controversial, primarily due to cost-effectiveness. The higher cost is the main disadvantage for patients undergoing robotic gastrectomy. In a report from Korea, the total cost difference between robotic and laparoscopic surgery was US$4490 or more [14]. The proportion of the cost paid by patients was even higher. Currently, the benefits of robotic over laparoscopic gastrectomy do not justify the higher cost of the robotic procedure [34]. In addition, the higher cost of the robotic gastrectomy procedure hinders the execution of randomized trials. However, the issue of cost, which is inflated due to instrument depreciation and maintenance, could be reduced if competing surgical robotic systems were made available [14].

### 3.5. Learning Curve

The learning curve of robotic gastrectomy is known to be shorter compared with that of laparoscopic surgery. However, most studies reporting on the learning curve of robotic gastrectomies enlisted experienced laparoscopic surgeons [20, 41]. Typically, 40–60 cases are needed to overcome the learning curve of laparoscopic gastrectomy [42–45], whereas a recent report showed that 12–14 cases were sufficient to overcome the learning curve of robotic surgery [46]. The shorter learning curve of robotic gastrectomy could allow even inexperienced surgeons to adopt the robotic technique easily and rapidly [47].

### 3.6. Lymph Node Dissection

D2 lymph node dissection is the recommended treatment for advanced gastric cancer [48–50]. Due to the technical difficulty and higher morbidity associated with D2 lymph node dissection, laparoscopic gastrectomy is typically restricted to cases of early gastric cancer. Dissecting around the suprapancreatic area is one of the most difficult parts in a laparoscopic gastrectomy. Comparative analyses between robotic and open gastrectomies reported no significant difference in the total number of retrieved lymph nodes [13]. Recent comparative studies between robotic and laparoscopic gastrectomies also showed no difference in the total number of retrieved lymph node [14, 25]. However, when evaluating the number of retrieved lymph nodes in the N2 area, robotic gastrectomy yielded more lymph nodes from this technically challenging area [6]. A thorough dissection in the N2 area is critical in cases of gastric cancer to improve oncological results [51]. Additionally, a comparative study of spleen-preserving total gastrectomies revealed that the mean numbers of retrieved lymph nodes in lymph node stations 10 and 11 were higher for robotic gastrectomy than laparoscopic gastrectomy (3.6 versus 1.9 retrieved N2 area lymph nodes; *p* = .014) even though the total number of retrieved lymph nodes was similar between the two groups (47.2 versus 42.8 total retrieved lymph nodes; *p* = .210) [11]. The high-resolution 3-D images, articulated instruments with seven degrees of freedom, and tremor elimination offered in robotic systems could allow surgeons to meticulously retrieve lymph nodes around complicated vascular structures or vital organs. This result is important because a recent review of laparoscopic gastrectomy for gastric cancer showed that it could retrieve fewer numbers of lymph nodes compared with that of open gastrectomy [52].

### 3.7. Long-Term Outcome

Only a few studies investigated long-term outcomes of robotic gastrectomy for gastric cancer. Coratti et al. showed that 5-year overall survival after robotic gastrectomy was 100%, 84.6%, 76.9%, and 21.5% in pathological stages IA, IB, II, and III, respectively. In this study, 5-year overall survival for patients with stage I and II disease was acceptable. These patients also had a low incidence of tumor recurrence and cancer-related mortality. For more advanced stages (III-IV), long-term survival with robotic gastrectomy was unsurprisingly poor; however, the survival was comparable to that of open and laparoscopic gastrectomy [53]. Nakauchi et al. showed that 3-year overall survival following robotic gastrectomy was 94.7%, 90.9%, 89.5%, and 62.5% in pathological stages IA, IB, II, and III, respectively. These results were also comparable to those observed for laparoscopic gastrectomy (96.2%, 95.1%, 83.8%, and 64.8% in stage IA, IB, II, and III, respectively) [38]. Although these results were produced by retrospective analyses, they indicate that robotic gastrectomy for gastric cancer may be safe in terms of oncological outcomes.

## 4. Application for Technically Demanding Procedure

Recent studies investigated the benefits of robotic surgery in specific patient types, such as those with advanced gastric cancer, those needing total gastrectomy, those with high BMI, and those with remnant gastric cancer. For advanced gastric cancer surgery, robotic gastrectomy retrieved a larger number of N2 area lymph nodes, as mentioned previously. The importance of complete suprapancreatic lymph node dissection in advanced disease had been shown to improve oncological outcomes [54]. In the subgroup analysis of a multicenter prospective comparative study, the robotic gastrectomy group showed significantly lower estimated blood loss than the laparoscopic gastrectomy group after D2 lymph node dissection (98.9 ± 105.7 versus 140.5 ± 143.1; *p* = .021), whereas no difference in estimated blood loss was observed in patients that underwent less extensive lymph node dissection (96.5 ± 144.2 versus 82.6 ± 91.7; *p* = .365) [30]. In addition, a recent report showed that robotic gastrectomy is safe in terms of the incidence of postoperative pancreatic fistulas, compared with laparoscopic gastrectomy, following suprapancreatic lymph node dissection (10% versus 22.5%; *p* < .001) [55]. Suda et al. also demonstrated similar results regarding less postoperative pancreatic fistula after robotic gastrectomy than laparoscopic surgery (conventional laparoscopic group, pancreatic fistula grade I: II: IIIa, 4 : 4 : 19 versus robotic group, grade I: II: IIIa, 8 : 0 : 0) [40]. These results might be brought about by the aforementioned advantages of robotic systems, such as three-dimensional magnified view with high definition, tremor filtering, and motion scaling [56, 57]. Further well-designed studies should be conducted to investigate if there are real benefits to robotic gastrectomy compared with laparoscopy in advanced gastric cancer.

While the use of minimally invasive techniques to perform distal subtotal gastrectomies has increased for both early and advanced gastric cancer, its use for upper gastric cancer has increased at a slower rate. It is the favored method in 49% of early gastric cancer cases and 6% of advanced gastric cancer cases due to the intrinsic difficulty in performing a total gastrectomy [58]. Several studies used robotic systems to perform total gastrectomies in upper gastric cancer patients and demonstrated acceptable short-term outcomes [59, 60]. Yoon et al. showed that the robotic total gastrectomy group had a longer operation time (305.8 ± 115.8 versus 210.2 ± 57.7 min; *p* < .001) but similar numbers of retrieved lymph node (42.8 ± 12.7 versus 39.4 ± 13.4; *p* = .209) and postoperative complications (16.7% versus 15.4) compared with the laparoscopic total gastrectomy group [61]. In a comparative study of robotic and laparoscopic spleen-preserving total gastrectomy, no difference in short-term outcomes was observed; however, there were slightly larger numbers of lymph nodes removed from specific areas, as mentioned above [11]. Robotic surgery could potentially be favored for technically demanding procedures [40, 62].

Because obesity is a risk factor for postoperative complications, conducting minimally invasive surgery in obese patients with gastric cancer can be challenging [63]. Lee et al. suggested that robotic surgery was better than laparoscopic gastrectomy with respect to the rate of adequately retrieved lymph nodes (more than 15 retrieved lymph nodes) in high BMI patients [25]. However, a recent report of a multicenter prospective study did not observe additional benefits using robotic procedures for obese patients compared with laparoscopic surgery [30]. Thus, the impact of robotic surgery in obese patients remains controversial.

One study investigated the use of a robotic system for remnant gastric cancer. Kwon et al. compared open and minimally invasive surgical techniques, including robotic surgery, for remnant gastric cancer. This study showed that completion total gastrectomy for remnant gastric cancer using minimally invasive techniques, including eight robotic cases, resulted in improved short-term outcomes and comparable oncological results compared with open surgery. For surgeons experienced in the techniques, the robotic approach could be decent option for managing remnant gastric cancer [64].

## 5. Future Applications for Robotic Systems in Gastric Cancer Treatment

Recently, image-guided surgery was introduced in the field of surgical oncology. Using infrared cameras installed on current robotic systems, fluorescent images can be incorporated into the surgical view [65]. Robotic systems can produce reconstructed vascular images and intraoperative endoscopic and radiologic images for the surgeon, reducing unwanted organ or vascular injury during gastrectomy [66]. Reduced port or single incision surgery for gastric cancer has been reported for laparoscopic gastrectomy [67, 68]. These procedures showed acceptable and feasible outcomes when performed by experienced gastric surgeons who had sufficient previous experience with conventional laparoscopic gastrectomy [67]. Recently, our institution reported the results of a phase I/II clinical trial of reduced port robotic gastrectomy, finding it to be a safe and feasible operation for early gastric cancer [69]. We have confidence that the advantages provided by a robotic surgical system could make these challenging procedures more comfortable to perform. So far, there is no solid evidence that robotic surgery can expand the indications of minimally invasive gastrectomy; however, based on our experiences and in light of emerging evidence, robotic surgery holds a greater possibility of being accepted for technically demanding procedures for treating gastric cancer than laparoscopic surgery.

## 6. Conclusion

The superiority of robotic procedures over conventional laparoscopic gastrectomy has not been proven at present. Despite the technical advantages of robotic surgery, its cost-effectiveness remains a major drawback. In the future, well-designed randomized trials with large sample sizes are needed to provide answers to controversial issues regarding the use of robotic systems for treating gastric cancer.

## Figures and Tables

**Table 1:** Summary of the latest case series for robotic gastrectomy.

Author	Year	Number of patients	Type of surgery (TG/STG)	LN dissection (limited/D2)	Operation time (min)	Blood loss (ml)	Number of retrieved LN	Hospital stay (days)	Morbidity (%)
Anderson et al. [70]	2007	7	0/7	—	420	300	24	4	14.3
Patriti et al. [71]	2008	13	4/−9	0/13	286	103	28.1	11.2	46.2
Song et al. [8]	2009	100	33/67	58/42	231.3	128.2	36.7	7.8	14
Hur et al. [72]	2010	7	2/−5	7/0	205	—	36	9	14
Liu et al. [73]	2010	9	5/−2	—	150–440	10–100	19–24(D1)/28–38(D2)	—	11
Isogaki et al. [74]	2011	61	14/46	22/39	250(TG)/388(STG)	150(TG)/61.8(STG)	43(TG)/42(STG)	13.3	4
D'Annibale et al. [9]	2011	24	11/13	0/24	267.5	30	28	6	8
Lee et al. [75]	2012	12	0/12	12/0	253	135	46	6.6	8
Yu et al. [76]	2012	41	12/29	—	285(TG)/225(STG)	180(TG)/150(STG)	34.2	—	5
Uyama et al. [77]	2012	25	0/25	7/18	361	51.8	44.3	12.1	11.2
Jiang et al. [78]	2012	120	—	—	245	70	22.5	6.3	5
Park et al. [19]	2013	200	46/154	—	248.8	146.1	37.9	8	10
Liu et al. [79]	2013	104	54(TG)/38(STG)/12(PG)	—	302.5(TG)/264.8(STG)/233.2(PG)	80.8	23.1	6.2	11.5
Tokunaga et al. [17]	2014	18	0/18	18/0	311.5	32.5	40	8	22.2
Barchi et al. [59]	2015	6	6/0	0/6	408		40	—	—
Coratti et al. [53]	2015	98	38/60		296.1	105.4	30.6	7	12.2
Parisi et al. [60]	2015	22	22/0	0/22	270	200	19.2	5.5	0
Quijano et al. [80]	2016	17	16/1	6/11	498	400	21	14.5	23.5

TG: total gastrectomy; STG: subtotal gastrectomy; PG: proximal gastrectomy.

**Table 2:** Summary of the latest comparative studies for robotic gastrectomy.

Author	Year	Type of approach	Number of patients	Type of surgery (TG/STG)	LN dissection (limited/D2)	Operation time (min)	Blood loss (ml)	Number of retrieved LN	Hospital stay (days)	Morbidity (%)	Mortality (%)
Pugliese et al. [81]	2009	R	9	0/9	0/9	350	92	27.5	11	—	—
		L	46	0/46	0/46	236	156	31.5	10	—	—
Song et al. [82]	2009	R, initial	20	0/20	16/4	230	94.8	35.3	5.7	5	0
		L, initial	20	0/20	10/−10	289	—	31.5	7.7	5	0
		L, recent	20	0/20	12/8	134	39.5	42.7	6.2	10	0
Pugliese et al. [83]	2010	R	16	0/16	0/18	344	90	25	10	6	—
		L	48	0/48	0/52	235	148	31	10	12.5	—
Kim et al. [39]	2010	R	16	0/16	2/14	259.2	30.3	41.1	5.1	0	0
		L	11	0/11	3/8	203.9	44.7	37.4	6.5	9	0
		O	12	0/12	0/12	126.7	78.8	43.3	6.7	16	—
Woo et al. [12]	2011	R	236	62/172	131/105	219.5	91.6	39	7.7	11	0.4
		L	591	108/481	312/279	170.7	147.9	37.4	7	13.7	0.3
Caruso et al. [10]	2011	R	29	12/17	0/29	290	197.6	28	9.6	41.4	0
		O	120	37/83	0/120	222	386.1	317	13.4	42.5	3.3
Yoon et al. [61]	2012	R	36	36/0	—	305.8	—	42.8	8.8	16.7	0
		L	65	65/0	—	210.2	—	39.4	10.3	15.4	0
Eom et al. [18]	2012	R	30	0/30	10/−20	229.1	152.8	30.2	7.9	13	0
		L	62	0/62	28/34	189.4	88.3	33.4	7.8	6	0
Huang et al. [20]	2012	R	39	7/32	5/34	430	50	32	7	14.4	2.6
		L	64	7/57	52/12	350	100	26	11	15.6	2.6
		O	586	179/407	70/516	320	400	34	12	14.7	1.4
Kang et al. [84]	2012	R	100	16/84	32/68	202	93.2	—	9.8	14	0
		L	282	37/245	—	173	173.4	—	8.1	10.3	0
Kim et al. [85]	2012	R	436	109/327		226	85	40.2	7.5	9.6	0.4
		L	861	158/703		176	112	37.6	7.8	8.9	0.3
		O	4542	1232/3309		158	192	40.5	10.2	10.1	0.4
Park et al. [34]	2012	R	30			218	60	35	7	17	0
		L	120			140	75	34	7	7.5	0
Hyun et al. [21]	2013	R	38	9/29	24/14	234.4	131.3	32.8	10.5	47.3	0
		L	83	18/65	65/18	220	130.4	32.6	11.9	38.5	0
Huang et al. [22]	2014	R	72	8/64	5/67	357.9	79.6	30.6	11	12.5	1.4
		L	73	10/63	32/41	319.8	116	28.1	13.2	8.2	1.4
Junfeng et al. [23]	2014	R	120	26(TG)/ 92(STG)/ 2(PG)		234.8	118.3	34.6	7.8	5.8	—
		L	394	118(TG)/ 261(STG)/ 15(PG)		221.3	137.6	32.7	7.9	4.3	—
Kim et al. [24]	2014	R	172		98/74	206.4	59.8	37.3	7.1	5.2	0
		L	481		246/235	167.1	134.9	36.8	6.7	4.2	3
Son et al. [11]	2014	R	51	51/0	0/51	264.1	163.4	47.2	8.6	15.7	2
		L	58	58/0	0/58	210.3	210.7	42.8	7.9	22.4	0
Noshiro et al. [37]	2014	R	21	0/21	13/8	439	96	44	8	9.5	0
		L	160	0/160	79/81	315	115	40	13	10	0
Han et al. [35]	2015	R	68	68(PPG)		258.3		33.4		19.1	0
		L	68	68(PPG)		193.9		36.5		22.1	0
Lee et al. [25]	2015	R	133	0/133	0/133	217.5	47	41.2	6.2	10.5	0
		L	267	0/267	0/267	171	87.1	39.9	7	12.7	0
Okumura et al. [36]	2015	R	49(E) 321(Y)	10(E)85(Y)/ 39(E)236(Y)	28(E)185(Y)/ 21(E)136(Y)	227.3(E) 219.6(Y)	84.8(E) 71.4(Y)	36.5(E) 41.5(Y)	5(E)5(Y)	14.3(E) 11.8(Y)	0(E)0(Y)
		L	132(E)	20/112	15/57	174.3	156.7	32.6	6	18.2	0.8
Seo et al. [55]	2015	R	40	0/40	22/18	243	76	40.4	6.75	27.5	0
		L	40	0/40	23/17	224	227	35.4	7.37	30	0
You et al. [86]	2015	R	16	0/16	5/11	271.9		44.3	11.4	12.5	0
		L	20	0/20	11/9	241		39.9	9.7	15	0
		O	12	0/12	11/1	178.8		41.6	11.8	8.3	0
Kim et al. [14]	2016	R	223	42(TG)/ 1(PG)/ 20(PPG)/ 160(STG)	113/110	226	50	33	6	30	0
		L	211	30(TG)/ 1(PG)/ 13(PPG)/ 167(STG)	128/83	180	60	32	6	30	0
Kim et al. [6]	2016	R	87	0/87	8/79	248.4	—	37.1	6.7	5.7	1.1
		L	288	0/288	95/193	230	—	34.1	7.4	9	0.3
Nakauchi et al. [38]	2016	R	84	27/57	35/49	378	44	40	14	2.4	—
		L	437	136/301	232/205	361	33	38	15	12.8	—
Shen et al. [26]	2016	R	93	23/70	43/50	257.1	176.6	33	9.4	9.8	—
		L	330	75/255	100/230	226.2	212.5	31.3	10.6	10	—
Wang et al. [13]	2016	R	151	47/104	24/127	242.7	94.2	29.1	5.7	9.3	—
		O	145	53/92	21/124	192.4	152.8	30.1	6.4	10.3	—

TG: total gastrectomy; STG: subtotal gastrectomy; PPG: pylorus preserving gastrectomy; PG: proximal gastrectomy.

## References

[1] Huscher C. G., Mingoli A., Sgarzini G. (2005). Laparoscopic versus open subtotal gastrectomy for distal gastric cancer: five-year results of a randomized prospective trial. *Annals of Surgery*.

[2] Kim Y. W., Baik Y. H., Yun Y. H. (2008). Improved quality of life outcomes after laparoscopy-assisted distal gastrectomy for early gastric cancer: results of a prospective randomized clinical trial. *Annals of Surgery*.

[3] Kim H. H., Hyung W. J., Cho G. S. (2010). Morbidity and mortality of laparoscopic gastrectomy versus open gastrectomy for gastric cancer: an interim report—a phase III multicenter, prospective, randomized trial (KLASS trial). *Annals of Surgery*.

[4] Strong V. E. (2014). Defining the role of laparoscopic gastrectomy for gastric cancer. *Journal of Clinical Oncology*.

[5] Jiang L., Yang K. H., Guan Q. L. (2013). Laparoscopy-assisted gastrectomy versus open gastrectomy for resectable gastric cancer: an update meta-analysis based on randomized controlled trials. *Surgical Endoscopy*.

[6] Kim Y. W., Reim D., Park J. Y. (2016). Role of robot-assisted distal gastrectomy compared to laparoscopy-assisted distal gastrectomy in suprapancreatic nodal dissection for gastric cancer. *Surgical Endoscopy*.

[7] Hashizume M., Sugimachi K. (2003). Robot-assisted gastric surgery. *The Surgical Clinics of North America*.

[8] Song J., Oh S. J., Kang W. H., Hyung W. J., Choi S. H., Noh S. H. (2009). Robot-assisted gastrectomy with lymph node dissection for gastric cancer: lessons learned from an initial 100 consecutive procedures. *Annals of Surgery*.

[9] D’Annibale A., Pende V., Pernazza G. (2011). Full robotic gastrectomy with extended (D2) lymphadenectomy for gastric cancer: surgical technique and preliminary results. *The Journal of Surgical Research*.

[10] Caruso S., Patriti A., Marrelli D. (2011). Open vs robot-assisted laparoscopic gastric resection with D2 lymph node dissection for adenocarcinoma: a case-control study. *The International Journal of Medical Robotics+Computer Assisted Surgery*.

[11] Son T., Lee J. H., Kim Y. M., Kim H. I., Noh S. H., Hyung W. J. (2014). Robotic spleen-preserving total gastrectomy for gastric cancer: comparison with conventional laparoscopic procedure. *Surgical Endoscopy*.

[12] Woo Y., Hyung W. J., Pak K. H. (2011). Robotic gastrectomy as an oncologically sound alternative to laparoscopic resections for the treatment of early-stage gastric cancers. *Archives of Surgery*.

[13] Wang G., Jiang Z., Zhao J. (2016). Assessing the safety and efficacy of full robotic gastrectomy with intracorporeal robot-sewn anastomosis for gastric cancer: a randomized clinical trial. *Journal of Surgical Oncology*.

[14] Kim H. I., Han S. U., Yang H. K. (2016). Multicenter prospective comparative study of robotic versus laparoscopic gastrectomy for gastric adenocarcinoma. *Annals of Surgery*.

[15] Delaney C. P., Lynch A. C., Senagore A. J., Fazio V. W. (2003). Comparison of robotically performed and traditional laparoscopic colorectal surgery. *Diseases of Colon & Rectum*.

[16] Giulianotti P. C., Coratti A., Angelini M. (2003). Robotics in general surgery: personal experience in a large community hospital. *Archives of Surgery*.

[17] Tokunaga M., Sugisawa N., Kondo J. (2014). Early phase II study of robot-assisted distal gastrectomy with nodal dissection for clinical stage IA gastric cancer. *Gastric Cancer*.

[18] Eom B. W., Yoon H. M., Ryu K. W. (2012). Comparison of surgical performance and short-term clinical outcomes between laparoscopic and robotic surgery in distal gastric cancer. *European Journal of Surgical Oncology*.

[19] Park J. Y., Kim Y. W., Ryu K. W., Eom B. W., Yoon H. M., Reim D. (2013). Emerging role of robot-assisted gastrectomy: analysis of consecutive 200 cases. *Journal of Gastric Cancer*.

[20] Huang K. H., Lan Y. T., Fang W. L. (2012). Initial experience of robotic gastrectomy and comparison with open and laparoscopic gastrectomy for gastric cancer. *Journal of Gastrointestinal Surgery*.

[21] Hyun M. H., Lee C. H., Kwon Y. J. (2013). Robot versus laparoscopic gastrectomy for cancer by an experienced surgeon: comparisons of surgery, complications, and surgical stress. *Annals of Surgical Oncology*.

[22] Huang K. H., Lan Y. T., Fang W. L. (2014). Comparison of the operative outcomes and learning curves between laparoscopic and robotic gastrectomy for gastric cancer. *PLoS One*.

[23] Junfeng Z., Yan S., Bo T. (2014). Robotic gastrectomy versus laparoscopic gastrectomy for gastric cancer: comparison of surgical performance and short-term outcomes. *Surgical Endoscopy*.

[24] Kim H. I., Park M. S., Song K. J., Woo Y., Hyung W. J. (2014). Rapid and safe learning of robotic gastrectomy for gastric cancer: multidimensional analysis in a comparison with laparoscopic gastrectomy. *European Journal of Surgical Oncology*.

[25] Lee J., Kim Y. M., Woo Y., Obama K., Noh S. H., Hyung W. J. (2015). Robotic distal subtotal gastrectomy with D2 lymphadenectomy for gastric cancer patients with high body mass index: comparison with conventional laparoscopic distal subtotal gastrectomy with D2 lymphadenectomy. *Surgical Endoscopy*.

[26] Shen W., Xi H., Wei B. (2016). Robotic versus laparoscopic gastrectomy for gastric cancer: comparison of short-term surgical outcomes. *Surgical Endoscopy*.

[27] Chuan L., Yan S., Pei-Wu Y. (2015). Meta-analysis of the short-term outcomes of robotic-assisted compared to laparoscopic gastrectomy. *Minimally Invasive Therapy & Allied Technologies*.

[28] Liu G., Shen W., Chen L., Wei B. (2016). Robotic versus laparoscopic gastrectomy for gastric cancer: a meta-analysis. *Zhonghua Wei Chang Wai Ke Za Zhi*.

[29] Son T., Hyung W. J. (2015). Robotic gastrectomy for gastric cancer. *Journals of Surgical Oncology*.

[30] Park J. M., Kim H. I., Han S. U. (2016). Who may benefit from robotic gastrectomy?: a subgroup analysis of multicenter prospective comparative study data on robotic versus laparoscopic gastrectomy. *European Journal of Surgical Oncology*.

[31] Han T. S., Kong S. H., Lee H. J. (2011). Dissemination of free cancer cells from the gastric lumen and from perigastric lymphovascular pedicles during radical gastric cancer surgery. *Annals of Surgical Oncology*.

[32] Marutsuka T., Shimada S., Shiomori K. (2003). Mechanisms of peritoneal metastasis after operation for non-serosa-invasive gastric carcinoma: an ultrarapid detection system for intraperitoneal free cancer cells and a prophylactic strategy for peritoneal metastasis. *Clinical Cancer Research*.

[33] Ojima T., Iwahashi M., Nakamori M. (2009). Association of allogeneic blood transfusions and long-term survival of patients with gastric cancer after curative gastrectomy. *Journal of Gastrointestinal Surgery*.

[34] Park J. Y., Jo M. J., Nam B. H. (2012). Surgical stress after robot-assisted distal gastrectomy and its economic implications. *The British Journal of Surgery*.

[35] Han D. S., Suh Y. S., Ahn H. S. (2015). Comparison of surgical outcomes of robot-assisted and laparoscopy-assisted pylorus-preserving gastrectomy for gastric cancer: a propensity score matching analysis. *Annals of Surgical Oncology*.

[36] Okumura N., Son T., Kim Y. M. (2016). Robotic gastrectomy for elderly gastric cancer patients: comparisons with robotic gastrectomy in younger patients and laparoscopic gastrectomy in the elderly. *Gastric Cancer*.

[37] Noshiro H., Ikeda O., Urata M. (2014). Robotically-enhanced surgical anatomy enables surgeons to perform distal gastrectomy for gastric cancer using electric cautery devices alone. *Surgical Endoscopy*.

[38] Nakauchi M., Suda K., Susumu S. (2016). Comparison of the long-term outcomes of robotic radical gastrectomy for gastric cancer and conventional laparoscopic approach: a single institutional retrospective cohort study. *Surgical Endoscopy*.

[39] Kim M. C., Heo G. U., Jung G. J. (2010). Robotic gastrectomy for gastric cancer: surgical techniques and clinical merits. *Surgical Endoscopy*.

[40] Suda K., Man-i M., Ishida Y., Kawamura Y., Satoh S., Uyama I. (2015). Potential advantages of robotic radical gastrectomy for gastric adenocarcinoma in comparison with conventional laparoscopic approach: a single institutional retrospective comparative cohort study. *Surgical Endoscopy*.

[41] Park S. S., Kim M. C., Park M. S., Hyung W. J. (2012). Rapid adaptation of robotic gastrectomy for gastric cancer by experienced laparoscopic surgeons. *Surgical Endoscopy*.

[42] Kim M. C., Jung G. J., Kim H. H. (2005). Learning curve of laparoscopy-assisted distal gastrectomy with systemic lymphadenectomy for early gastric cancer. *World Journal of Gastroenterology*.

[43] Hu W. G., Ma J. J., Zang L. (2014). Learning curve and long-term outcomes of laparoscopy-assisted distal gastrectomy for gastric cancer. *Journal of Laparoendoscopic & Advanced Surgical Techniques*.

[44] Kunisaki C., Makino H., Yamamoto N. (2008). Learning curve for laparoscopy-assisted distal gastrectomy with regional lymph node dissection for early gastric cancer. *Surgical Laparoscopy, Endoscopy & Percutaneous Techniques*.

[45] Jin S. H., Kim D. Y., Kim H. (2007). Multidimensional learning curve in laparoscopy-assisted gastrectomy for early gastric cancer. *Surgical Endoscopy*.

[46] Zhou J., Shi Y., Qian F. (2015). Cumulative summation analysis of learning curve for robot-assisted gastrectomy in gastric cancer. *Journal of Surgical Oncology*.

[47] Jayaraman S., Quan D., Al-Ghamdi I., El-Deen F., Schlachta C. M. (2010). Does robotic assistance improve efficiency in performing complex minimally invasive surgical procedures?. *Surgical Endoscopy*.

[48] Ajani J. A., Bentrem D. J., Besh S. (2013). Gastric cancer, version 2.2013: featured updates to the NCCN guidelines. *Journal of the National Comprehesive Cancer Network*.

[49] Japanese Gastric Cancer Association (2016). Japanese gastric cancer treatment guidelines 2014 (ver. 4). *Gastric Cancer*.

[50] Lee J. H., Kim J. G., Jung H. K. (2014). Synopsis on clinical practice guideline of gastric cancer in Korea: an evidence-based approach. *The Korean Journal of Gastroenterology*.

[51] Songun I., Putter H., Kranenbarg E. M., Sasako M., van de Velde C. J. (2010). Surgical treatment of gastric cancer: 15-year follow-up results of the randomised nationwide Dutch D1D2 trial. *The Lancet Oncology*.

[52] Viñuela E. F., Gonen M., Brennan M. F., Coit D. G., Strong V. E. (2012). Laparoscopic versus open distal gastrectomy for gastric cancer: a meta-analysis of randomized controlled trials and high-quality nonrandomized studies. *Annals of Surgery*.

[53] Coratti A., Fernandes E., Lombardi A. (2015). Robot-assisted surgery for gastric carcinoma: five years follow-up and beyond: a single western center experience and long-term oncological outcomes. *European Journal of Surgical Oncology*.

[54] Hartgrink H. H., Van de Velde C. J., Putter H. (2004). Extended lymph node dissection for gastric cancer: who may benefit? Final results of the randomized Dutch gastric cancer group trial. *Journal of Clinical Oncology*.

[55] Seo H. S., Shim J. H., Jeon H. M., Park C. H., Song K. Y. (2015). Postoperative pancreatic fistula after robot distal gastrectomy. *The Journal of Surgical Research*.

[56] Suda K., Nakauchi M., Inaba K., Ishida Y., Uyama I. (2015). Revising robotic surgery for stomach, potential benefits revised II: prevention of pancreatic fistula. *Translational Gastrointestinal Cancer*.

[57] Suda K., Nakauchi M., Inaba K., Ishida Y., Uyama I. (2016). Robotic surgery for upper gastrointestinal cancer: current status and future perspectives. *Digestive Endoscopy*.

[58] Brenkman H. J., Haverkamp L., Ruurda J. P., van Hillegersberg R. (2016). Worldwide practice in gastric cancer surgery. *World Journal of Gastroenterology*.

[59] Barchi L. C., Jacob C. E., Franciss M. Y., Kappaz G. T., Rodrigues Filho E. D., Zilberstein B. (2016). Robotic digestive tract reconstruction after total gastrectomy for gastric cancer: a simple way to do it. *The International Journal of Medical Robotic+Computer Assited Surgery*.

[60] Parisi A., Ricci F., Trastulli S. (2015). Robotic total gastrectomy with intracorporeal robot-sewn anastomosis: a novel approach adopting the double-loop reconstruction method. *Medicine (Baltimore)*.

[61] Yoon H. M., Kim Y. W., Lee J. H. (2012). Robot-assisted total gastrectomy is comparable with laparoscopically assisted total gastrectomy for early gastric cancer. *Surgical Endoscopy*.

[62] Terashima M., Tokunaga M., Tanizawa Y. (2015). Robotic surgery for gastric cancer. *Gastric Cancer*.

[63] Shin H. J., Son S. Y., Cui L. H. (2015). Is there any role of visceral fat area for predicting difficulty of laparoscopic gastrectomy for gastric cancer?. *Journal of Gastric Cancer*.

[64] Kwon I. G., Cho I., Guner A. (2014). Minimally invasive surgery for remnant gastric cancer: a comparison with open surgery. *Surgical Endoscopy*.

[65] Herrell S. D., Galloway R. L., Su L. M. (2012). Image-guided robotic surgery: update on research and potential applications in urologic surgery. *Current Opinion in Urology*.

[66] Kim Y. M., Baek S. E., Lim J. S., Hyung W. J. (2013). Clinical application of image-enhanced minimally invasive robotic surgery for gastric cancer: a prospective observational study. *Journal of Gastrointestinal Surgery*.

[67] Kunisaki C., Makino H., Yamaguchi N. (2016). Surgical advantages of reduced-port laparoscopic gastrectomy in gastric cancer. *Surgical Endoscopy*.

[68] Ahn S. H., Son S. Y., Jung D. H. (2015). Solo intracorporeal esophagojejunostomy reconstruction using a laparoscopic scope holder in single-port laparoscopic total gastrectomy for early gastric cancer. *Journal of Gastric Cancer*.

[69] Lee S., Kim J. K., Kim Y. N. (2017). Safety and feasibility of reduced-port robotic distal gastrectomy for gastric cancer: a phase I/II clinical trial. *Surgical Endoscopy*.

[70] Anderson C., Ellenhorn J., Hellan M., Pigazzi A. (2007). Pilot series of robot-assisted laparoscopic subtotal gastrectomy with extended lymphadenectomy for gastric cancer. *Surgical Endoscopy*.

[71] Patriti A., Ceccarelli G., Bellochi R. (2008). Robot-assisted laparoscopic total and partial gastric resection with D2 lymph node dissection for adenocarcinoma. *Surgical Endoscopy*.

[72] Hur H., Kim J. Y., Cho Y. K., Han S. U. (2010). Technical feasibility of robot-sewn anastomosis in robotic surgery for gastric cancer. *Journal of Laparoendoscopic & Advanced Surgical Techniques.*.

[73] Liu F. L., Lv C. T., Qin J. (2010). Da Vinci robot-assisted gastrectomy with lymph node dissection for gastric cancer: a case series of 9 patients. *Zhonghua Wei Chang Wai Ke Za Zhi*.

[74] Isogaki J., Haruta S., Man-i M. (2011). Robot-assisted surgery for gastric cancer: experience at our institute. *Pathobiology*.

[75] Lee H. H., Hur H., Jung H., Jeon H. M., Park C. H., Song K. Y. (2011). Robot-assisted distal gastrectomy for gastric cancer: initial experience. *American Journal of Surgery*.

[76] Yu P. W., Tang B., Zeng D. Z. (2012). Robotic-assisted radical gastrectomy using da Vinci robotic system: a report of 41 cases. *Zhonghua Wei Chang Wai Ke Za Zhi*.

[77] Uyama I., Kanaya S., Ishida Y., Inaba K., Suda K., Satoh S. (2012). Novel integrated robotic approach for suprapancreatic D2 nodal dissection for treating gastric cancer: technique and initial experience. *World Journal of Surgery*.

[78] Jiang Z. W., Zhao K., Wang G. (2012). Application of surgical robotic system in patients with gastric cancer: a report of 120 cases. *Zhonghua Wei Chang Wai Ke Za Zhi*.

[79] Liu X. X., Jiang Z. W., Chen P., Zhao Y., Pan H. F., Li J. S. (2013). Full robot-assisted gastrectomy with intracorporeal robot-sewn anastomosis produces satisfying outcomes. *World Journal of Gastroenterology*.

[80] Quijano Y., Vicente E., Ielpo B. (2016). Full robot-assisted gastrectomy: surgical technique and preliminary experience from a single center. *Journal of Robotic Surgery*.

[81] Pugliese R., Maggioni D., Sansonna F. (2009). Outcomes and survival after laparoscopic gastrectomy for adenocarcinoma. Analysis on 65 patients operated on by conventional or robot-assisted minimal access procedures. *European Journal of Surgical Oncology*.

[82] Song J., Kang W. H., Oh S. J., Hyung W. J., Choi S. H., Noh S. H. (2009). Role of robotic gastrectomy using da Vinci system compared with laparoscopic gastrectomy: initial experience of 20 consecutive cases. *Surgical Endoscopy*.

[83] Pugliese R., Maggioni D., Sansonna F. (2010). Subtotal gastrectomy with D2 dissection by minimally invasive surgery for distal adenocarcinoma of the stomach: results and 5-year survival. *Surgical Endoscopy*.

[84] Kang B. H., Xuan Y., Hur H., Ahn C. W., Cho Y. K., Han S. U. (2012). Comparison of surgical outcomes between robotic and laparoscopic gastrectomy for gastric cancer: the learning curve of robotic surgery. *Journal of Gastric Cancer*.

[85] Kim K. M., An J. Y., Kim H. I., Cheong J. H., Hyung W. J., Noh S. H. (2012). Major early complications following open, laparoscopic and robotic gastrectomy. *The British Journal of Surgery*.

[86] You Y. H., Kim Y. M., Ahn D. H. (2015). Beginner surgeon's initial experience with distal subtotal gastrectomy for gastric cancer using a minimally invasive approach. *Journal of Gastric Cancer*.

